# Red Blood Cell Metabolism in Pyruvate Kinase Deficient Patients

**DOI:** 10.3389/fphys.2021.735543

**Published:** 2021-10-21

**Authors:** Micaela K. Roy, Francesca Cendali, Gabrielle Ooyama, Fabia Gamboni, Holmes Morton, Angelo D’Alessandro

**Affiliations:** ^1^Department of Biochemistry and Molecular Genetics, University of Colorado Denver – Anschutz Medical Campus, Aurora, CO, United States; ^2^Central Pennsylvania Clinic, A Medical Home for Special Children and Adults, Belleville, PA, United States

**Keywords:** red blood cells (erythrocytes), pyruvate kinase deficiency, metabolomics, oxidative stress, underrepresented minorities, rural America

## Abstract

**Background:** Pyruvate kinase deficiency (PKD) is the most frequent congenital enzymatic defect of glycolysis, and one of the most common causes of hereditary non spherocytic hemolytic anemia. Therapeutic interventions are limited, in part because of the incomplete understanding of the molecular mechanisms that compensate for the metabolic defect.

**Methods:** Mass spectrometry-based metabolomics analyses were performed on red blood cells (RBCs) from healthy controls (*n*=10) and PKD patients (*n*=5).

**Results:** In PKD patients, decreases in late glycolysis were accompanied by accumulation of pentose phosphate pathway (PPP) metabolites, as a function of oxidant stress to purines (increased breakdown and deamination). Markers of oxidant stress included increased levels of sulfur-containing compounds (methionine and taurine), polyamines (spermidine and spermine). Markers of hypoxia such as succinate, sphingosine 1-phosphate (S1P), and hypoxanthine were all elevated in PKD subjects. Membrane lipid oxidation and remodeling was observed in RBCs from PKD patients, as determined by increases in the levels of free (poly-/highly-unsaturated) fatty acids and acyl-carnitines.

**Conclusion:** In conclusion, in the present study, we provide the first overview of RBC metabolism in patients with PKD. Though limited in scope, the study addresses the need for basic science to investigate pathologies targeting underrepresented minorities (Amish population in this study), with the ultimate goal to target treatments to health disparities.

## Introduction

Pyruvate kinase deficiency (PKD) is the most frequent congenital enzymatic defect of glycolysis, and one of the most common causes of hereditary non spherocytic hemolytic anemia (Zanella et al., 2007). The disease has diverse clinical presentations, with levels of hemolysis and the resultant anemia varying from patient to patient (Svidnicki et al., 2018; Enegela and Anjum, 2021). As such, clinical presentations range from mild and fully compensated anemia to more severe forms requiring repeated transfusion (Zanella et al., 2005). PKD is traditionally considered an autosomal recessive condition (Zanella et al., 2007). Due to the founder effect, PKD is more prevalent in specific groups including the Pennsylvania Amish and the Romani people (Rider et al., 2011).

A well-established hypothesis posits that common metabolic enzymopathies in humans, like PKD or glucose 6-phosphate dehydrogenase deficiency (Francis et al., 2020), may have arisen and were selected for as a result of evolutive pressure by endemic diseases – such as malaria (Ayi et al., 2008). By altering enzymatic activity at rate-limiting steps erythrocyte energy and redox metabolism, these mutations are deemed to confer resistance to parasitic infection at the erythrocytic stage (Ayi et al., 2008). Comprising over 80% of cells in the human body, red blood cells (RBCs) are involved in a transport system that brings oxygen and nutrients to the tissues (Nemkov et al., 2018a). Because of their ubiquity and movement throughout the body, they offer a unique lens into whole system metabolism (Nemkov et al., 2018a). Lacking a nucleus and organelles, red cells depend almost entirely on glycolysis for energy production. By constraining the rate of one of the two adenosine triphosphate (ATP)-generating steps in glycolysis, PKD dramatically alters RBC function, metabolism, and lifespan (Chapman and Schaumburg, 1967; Nathan et al., 1968; Delivoria-Papadopoulos et al., 1969). Specifically, pyruvate kinase is responsible for catalyzing the ATP producing conversion of phosphoenolpyruvate to pyruvate, a process responsible for the generation of 50% of all ATP in RBCs (Grace et al., 2015). Anemia in PKD patients is thought to occur as a result of reduced levels of ATP which are critical in maintaining processes required for cell function (Nathan et al., 1968), including fueling ion pumps, preserving membrane structural homeostasis and synthesizing key reducing equivalents such as glutathione (GSH; D’Alessandro et al., 2019). As ATP levels decrease, the energy-dependent sodium potassium ATPases slow, and potassium exits the cell membrane – a phenomenon that is particularly relevant in the context of common iatrogenic interventions, such as blood storage and transfusion (Yoshida et al., 2019). Alteration of sodium/potassium equilibria in the inner/extracellular compartment concomitantly impairs the function of ATP/sodium-dependent calcium pumps (Lew and Tiffert, 2017), triggering processes of erythrocyte-specific suicidal death (eryptosis; Nemkov et al., 2020a). Loss of ion homeostasis promotes water leaking out of the cell owing to the hypotonicity of the intracellular compartment, leading to dehydration, dysfunction, and removal of RBCs from the bloodstream (Van Wijk and Van Solinge, 2005). Vice versa, boosting PK activity in the context of hemoglobinopathies that alter RBC redox and energy metabolism (e.g., sickle cell disease) has been proposed as a viable strategy to prevent RBC lysis and untimely removal from the bloodstream (Rab et al., 2021).

Though the understanding of PKD has expanded in recent years, its full effects on RBC metabolism have not been fully described. Moreover, PKD is underdiagnosed in the general population, due in part to diagnostic gaps that arise from the disease’s heterogeneity (Bianchi et al., 2019). An understanding of the effects of this disorder on the metabolome may allow for better diagnosis, management, and treatment of this condition.

While previous reports focused on dried blood spot analysis of whole blood from PKD patients (Dooijeweert et al., 2021), in this study, we attempted to characterize the metabolome of RBCs from PKD patients. We hypothesized that PKD samples would show reprogramming of energy metabolism, with increases in early glycolytic metabolites and decreases in late glycolytic metabolites. We also predicted that PKD samples would display metabolic changes indicative of the systemic hypoxia that occurs in other anemias, but with less severe presentations due to the compensated nature of the disease.

## Materials and Methods

### Sample Collection

Samples were collected through venipuncture from 10 healthy controls and five patients with PKD at the Central Pennsylvania Clinic, at Boston Children’s and Lancaster General Hospitals under institutionally reviewed Pyruvate Kinase Deficiency Natural History Study Protocol No. 2014-12 and upon signing of informed consent. All patients were homozygous for the variant PKLR c.1436G>A; p.Arg479His; four female children from Amish families and one male age 60; all patients had been splenectomized. All patients manifested a compensated hemolytic anemia with hemoglobin levels of 9–11g/dl, hematocrit of 28–30% and high reticulocyte counts (20–40%). RBCs were separated from whole blood through centrifugation for 10min at 4°C and 2,000*g*.

### Metabolomics

Metabolomics analyses were performed as extensively described in previous studies (Nemkov et al., 2020b; Stefanoni et al., 2020; D’Alessandro et al., 2021). A volume of 50μl of frozen RBC aliquots was extracted in 450μl of methanol:acetonitrile:water (5:3:2, v/v/v). After vortexing at 4°C for 30min, extracts were separated from the protein pellet by centrifugation for 10min at 10,000*g* at 4°C and stored at −80°C until analysis. Ultra-High-Pressure Liquid Chromatography-Mass Spectrometry (UHPLC-MS) analyses were performed using a Vanquish UHPLC coupled online to a Q Exactive mass spectrometer (Thermo Fisher, Bremen, Germany). Samples were analyzed using a 5min gradient as described (Nemkov et al., 2017, 2019; Reisz et al., 2019). Solvents were supplemented with 0.1% formic acid for positive mode runs and 1mM ammonium acetate for negative mode runs. MS acquisition, data analysis, and elaboration was performed as described (Nemkov et al., 2017, 2019; Reisz et al., 2019).

### Statistical Methods

Graphs and statistical analyses (unpaired *t*-test) were prepared with GraphPad Prism 8.0 (GraphPad Software, Inc., La Jolla, CA, United States) and MetaboAnalyst 4.0 (Chong et al., 2018).

## Results

### RBCs From PKD Patients Show a Distinct Metabolic Fingerprint When Compared to Controls

**Figure 1:**
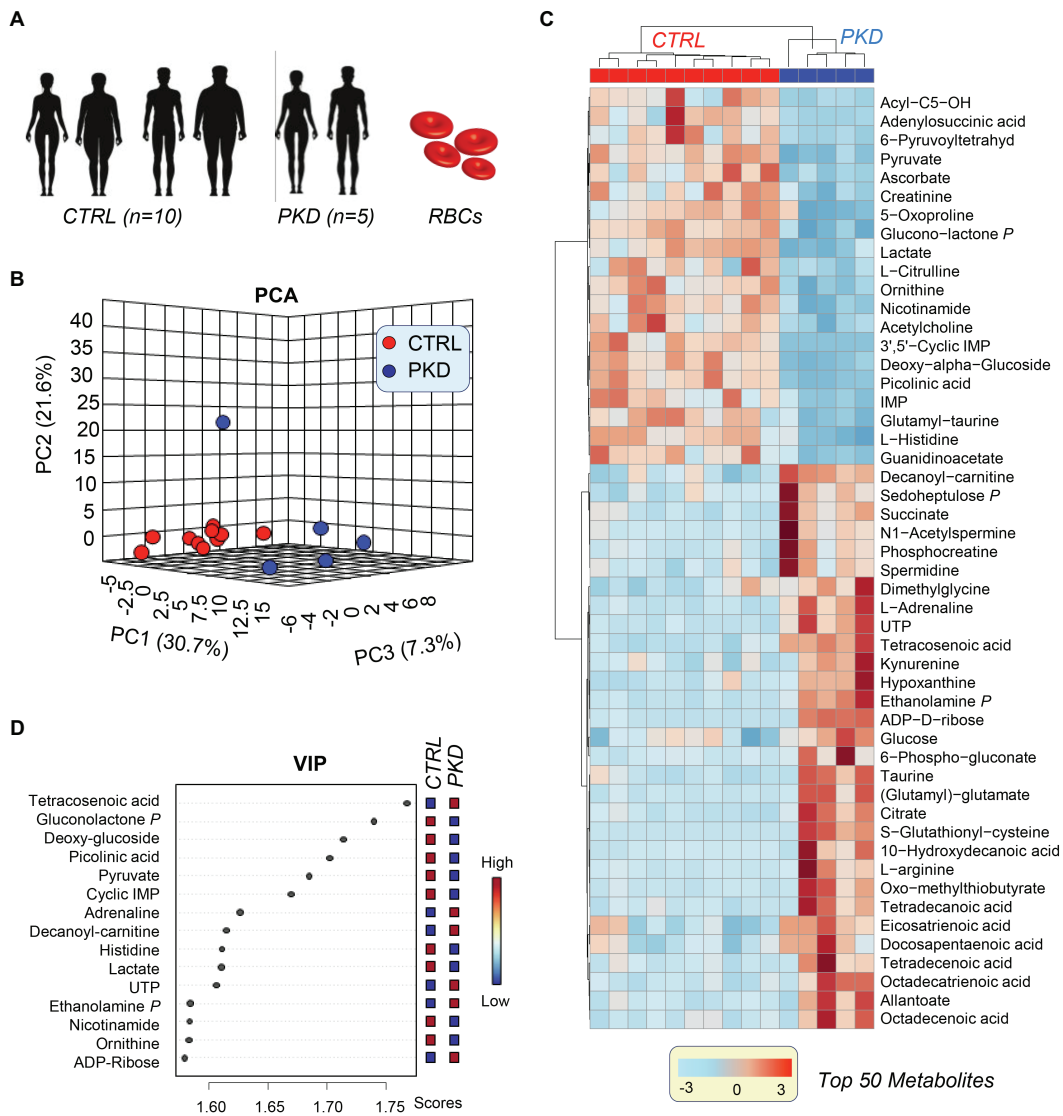
Multivariate analysis reveals a unique metabolic profile of pyruvate kinase deficiency (PKD) patients when compared with unaffected controls. **(A)** Samples from five PKD patients and 10 unaffected controls were analyzed with untargeted metabolomics procedures using ultra-high-pressure liquid chromatography-mass spectrometry (UHPLC-MS). **(B)** A principal component analysis (PCA) clustered samples by metabolic phenotype and revealed that PKD samples were metabolically divergent from control samples. **(C)** The variable importance in projection (VIP) values from a partial least square discriminant analysis (PLS-DA) display the metabolites that contributed most to the clustering pattern. **(D)** A hierarchal clustering analysis (HCA) of the top 50 significant metabolites by ANOVA shows differences between PKD and CTL samples, especially in amino acid, glucose, and fatty acid metabolism.

Metabolomics results from the analyses performed on samples collected from five PKD patients and 10 healthy controls (Figure 1A) are extensively reported in Supplementary Table 1. Unsupervised analyses of metabolomics data, including principal component analysis (PCA), hierarchical clustering analysis (HCA), and calculation of the variable importance in projection (VIP) from partial leas square-discriminant analyses (PLS-DA) are reported in Figures 1B–D, respectively. These analyses revealed a unique metabolic phenotype of PKD patients, with 30.7% of the total metabolic variance across all samples attributable to metabolites affected by the (PKD) condition (Figure 1B). The top 15 metabolites that contributed most to the distinct clustering pattern of PKD samples are listed in Figure 1D. These features included metabolites from glucose metabolism (especially late glycolysis/byproducts – pyruvate and lactate), as well as metabolites from other pathways including the pentose phosphate pathway (PPP) amino acid metabolism and purine metabolism. A more comprehensive list of differential metabolites is provided in the HCA in Figure 1C, which displays quantitative trends for the top 50 significant metabolites by ANOVA. This analysis highlighted additional differences between the two groups, including several intermediates of pathways such as sulfur-containing amino acid metabolism, glucose metabolism, and fatty acid metabolism (Figure 1D).

### Metabolomics Analyses Show Reduced Pyruvate Kinase Activity in PKD RBCs When Compared With Controls

**Figure 2:**
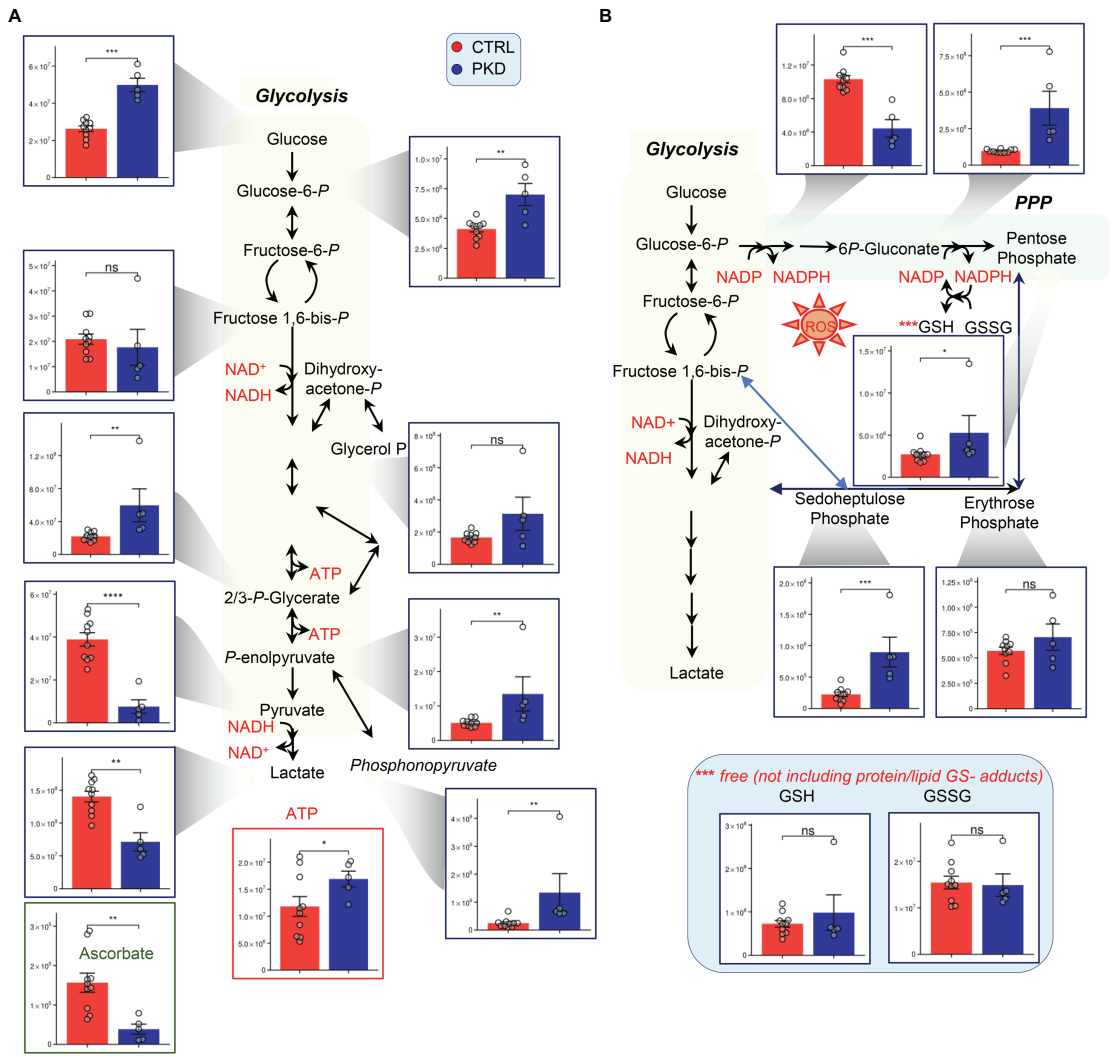
Pyruvate kinase deficiency reprograms **(A)** glycolysis and **(B)** the pentose phosphate pathway (PPP). Y-axes represent peak area (arbitrary units). Asterisks indicate significant results, error bars represent ±SEM (unpaired *t*-test, two-tailed distribution, ^*^p<0.05, ^**^p<0.01, ^***^p<0.001, and ^****^p<0.0001).

Consistent with compromised pyruvate kinase activity, PKD patients show significant accumulation of metabolites upstream of pyruvate kinase including Glucose, hexose phosphate isobars (H6P), 2,3-phosphoglycerate isomers, and phosphoenolpyruvate (Figure 2A). As predicted, analyses also revealed a significant depletion of products downstream of pyruvate kinase, including pyruvate and lactate. In the PPP, NADP+ was down in PKD patients and 6P-Gluconate, sedoheptulose phosphate, and pentose phosphate were increased, indicating a potential shift to the PPP (Figure 2B). In RBCs, PPP is the main contributor to NADPH synthesis, a reducing cofactor that is essential in maintaining the pool of reduced GSH, a metabolite crucial in mitigating oxidative damage (D’Alessandro et al., 2019). Despite the apparent increase in the fluxes through the PPP – as inferred from steady state levels of intermediates and products of the PPP – and the expected concomitant increase in NADPH production, the levels of GSH and GSSG in PKD patients were comparable to those of the controls. However, the low NAPD+ suggests that niacin may become rate limiting in some PKD patients. Another important readily supplemented anti-oxidant, ascorbate, was decreased in all PKD patients compared to controls. Vitamin-C deficiency may become especially important in patients with iron overload syndrome, which is common in the settling of transfusions for anemia as well as from chronic hemolysis and high red cell turnover driven by elevated erythropoietin concentrations, which are typical of PK deficiency.

Interestingly, ATP appeared to increase in PKD patients, despite the impairment of the ATP generating conversion from phosphoenolpyruvate to pyruvate, perhaps suggesting a compensatory mechanism – such as decreased consumption or increased fluxes through early ATP-generating steps, such as catabolism of 1,3-diphosphoglycerate and decreased fluxes through the Rapoport Luebering shunt. It is also likely that the increased ATP in the red cell mass reflects high reticulocyte numbers (as high as 40% in one of the subjects enrolled in this study) and continued O_2_-dependent ATP production in residual mitochondria. The influence of reticulocytes upon the metabolomic profile is also suggested by the increased citric acid cycle intermediates citrate and succinate.

Previous research has shown that PKD can trigger reticulocytosis especially in patients that undergo splenectomy (all of the PKD subjects in this study), increasing the supply of mitochondria-bearing immature RBCs to compensate for decreased ATP production in mature red cells (Nathan et al., 1968; Pekrun et al., 1995). In these reticulocytes, pyruvate can be derived from sources in addition to glycolysis, allowing for the generation of ATP through the Krebs cycle and electron transport chain (ETC; Nathan et al., 1968). Of note, significant decreases in late glycolytic metabolites downstream to PK, pyruvate, and lactate, was accompanied by significant increases in the levels of 2,3 DPG to pyruvate ratio and increases in phosphonopyruvate, a metabolite generated through alternative metabolism of phosphoenolpyruvate (Figure 2B). Increased red cell 2,3-BPG favorably shifts the oxygen dissociation curve to release oxygen to tissues, in part, compensating for anemia.

### Altered Levels of Carboxylic Acids, Glutaminolysis, and Branched Chain Amino Acids in PKD RBCs

**Figure 3:**
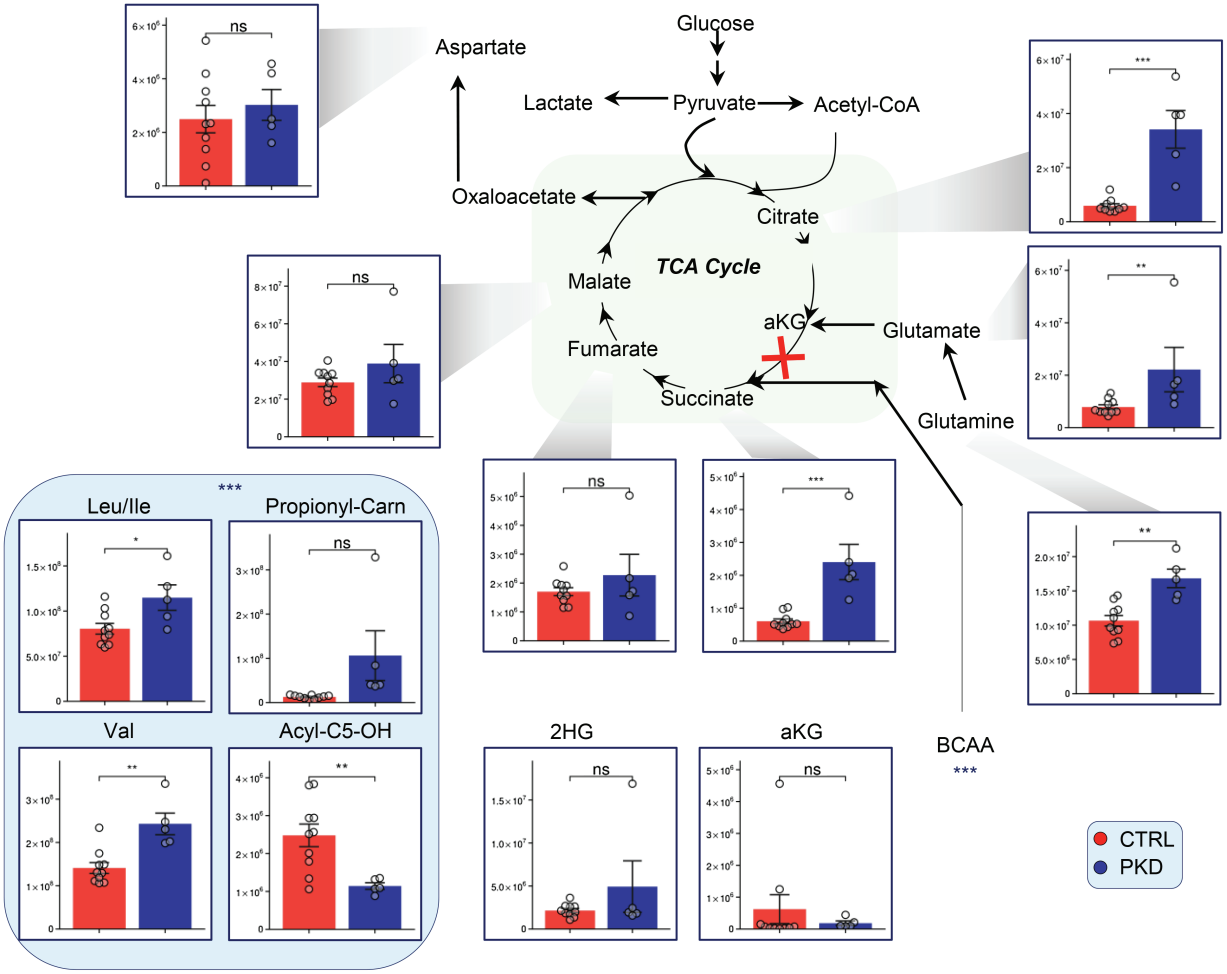
Pyruvate kinase deficiency remodels tricarboxylic acid cycle (TCA) metabolism and alters levels of branched-chain amino acid (BCAA) precursors in red blood cells (RBCs). Overview of the TCA and upstream metabolites including BCAA in PKD patients compared with controls. Y-axes represent peak area (arbitrary units). Asterisks indicate significant results, error bars represent ±SEM (unpaired *t*-test, two-tailed distribution, ^*^p<0.05, ^**^p<0.01, and ^***^p<0.001).

Metabolomics analyses revealed that levels of citrate and succinate were significantly elevated in PKD samples when compared with the controls (Figure 3). Additionally, the metabolic precursors to alpha ketoglutarate, glutamate, glutamine, and the branched chain amino acids – which can be metabolized to succinyl-CoA, were significantly elevated in PKD samples (Figure 3).

Interestingly, Acyl-C5-OH – a byproduct of branched chain amino acid catabolism – was significantly lower in PKD patients, potentially suggesting an increase in consumption of this metabolite through a different pathway.

### PKD RBCs Display Alterations of Methionine, Arginine, and Purine Metabolism

**Figure 4:**
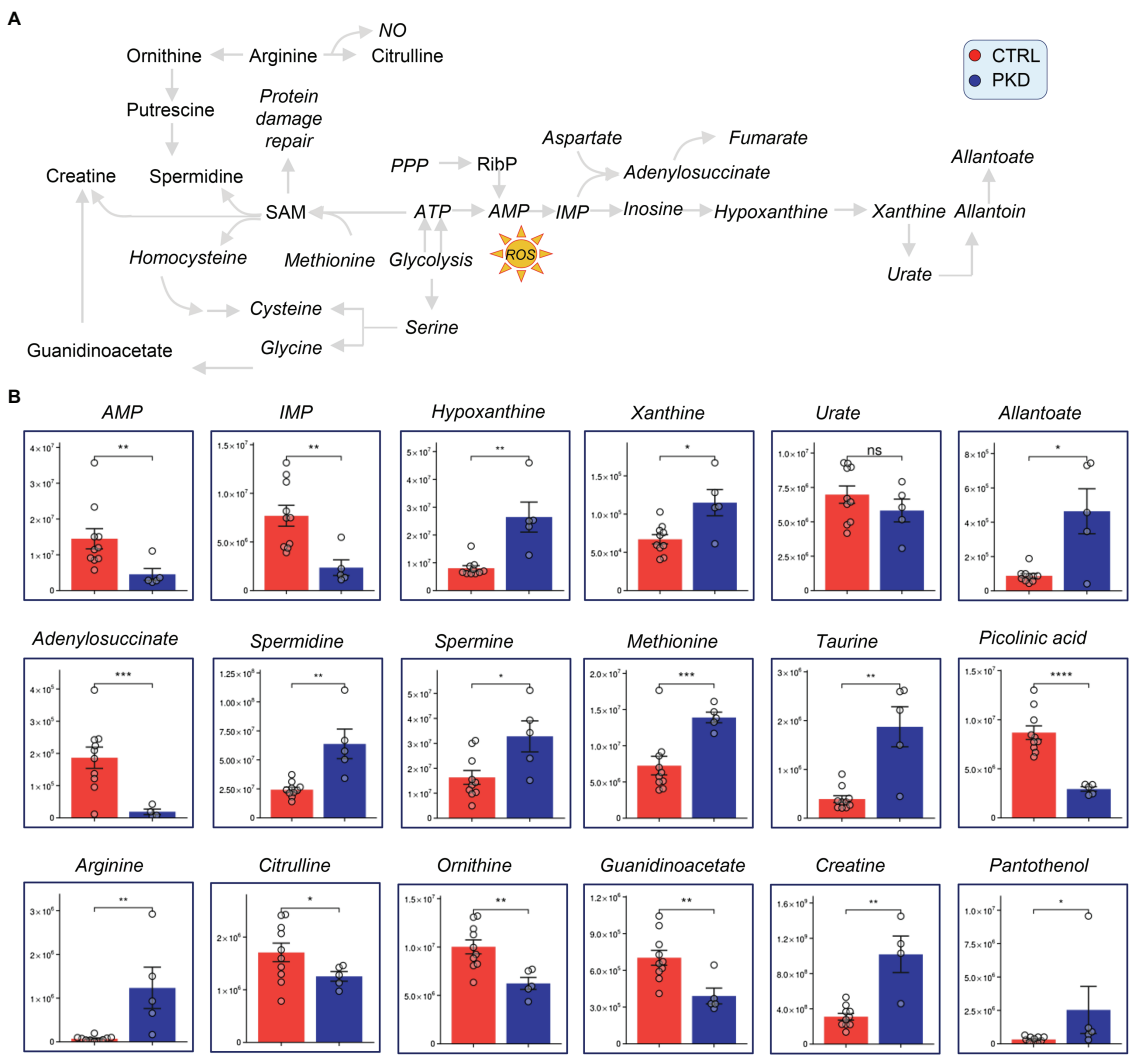
Pyruvate kinase deficiency patients show altered levels of polyamine, purine, methionine, and arginine metabolism. Overview of methionine, arginine, and purine metabolism, and downstream metabolites (A). (B) Y-axes represent peak area (arbitrary units). Asterisks indicate significant results, error bars represent ±SEM (unpaired *t*-test, two-tailed distribution, ^*^p<0.05, ^**^p<0.01, ^***^p<0.001, and ^****^p<0.0001).

Pyruvate kinase deficiency samples showed reduced levels of AMP, IMP, and adenylosuccinate suggesting that adenosine supplies may be limited or reflect increased oxidative stress as reflected in markers (Nemkov et al., 2018b), hypoxanthine, xanthine, and allantoate (Figure 4). Also, consistent with increased oxidant stress, comparably to that observed in patients with G6PD deficiency, we found an increase in oxidant stress-induced isoaspartyl damage (Ingrosso et al., 2002; D’Alessandro et al., 2021) and the levels of sulfur-containing antioxidant metabolites methionine and taurine were significantly increased in RBCs from PKD patients (Figure 4).

Although arginine was dramatically increased in PKD patients when compared with controls, its downstream catabolites citrulline and ornithine were significantly decreased in PKD samples (Figure 4). This could suggest that arginine is being channeled into a different pathway. Notably, the downstream products of ornithine catabolism and purine salvage pathway, including creatinine, spermidine, and spermine are also elevated (Figure 4), possibly indicating that arginine is getting converted through ornithine.

### Metabolomics Analyses Showed Elevated Levels of Fatty Acids in PKD Patients

**Figure 5:**
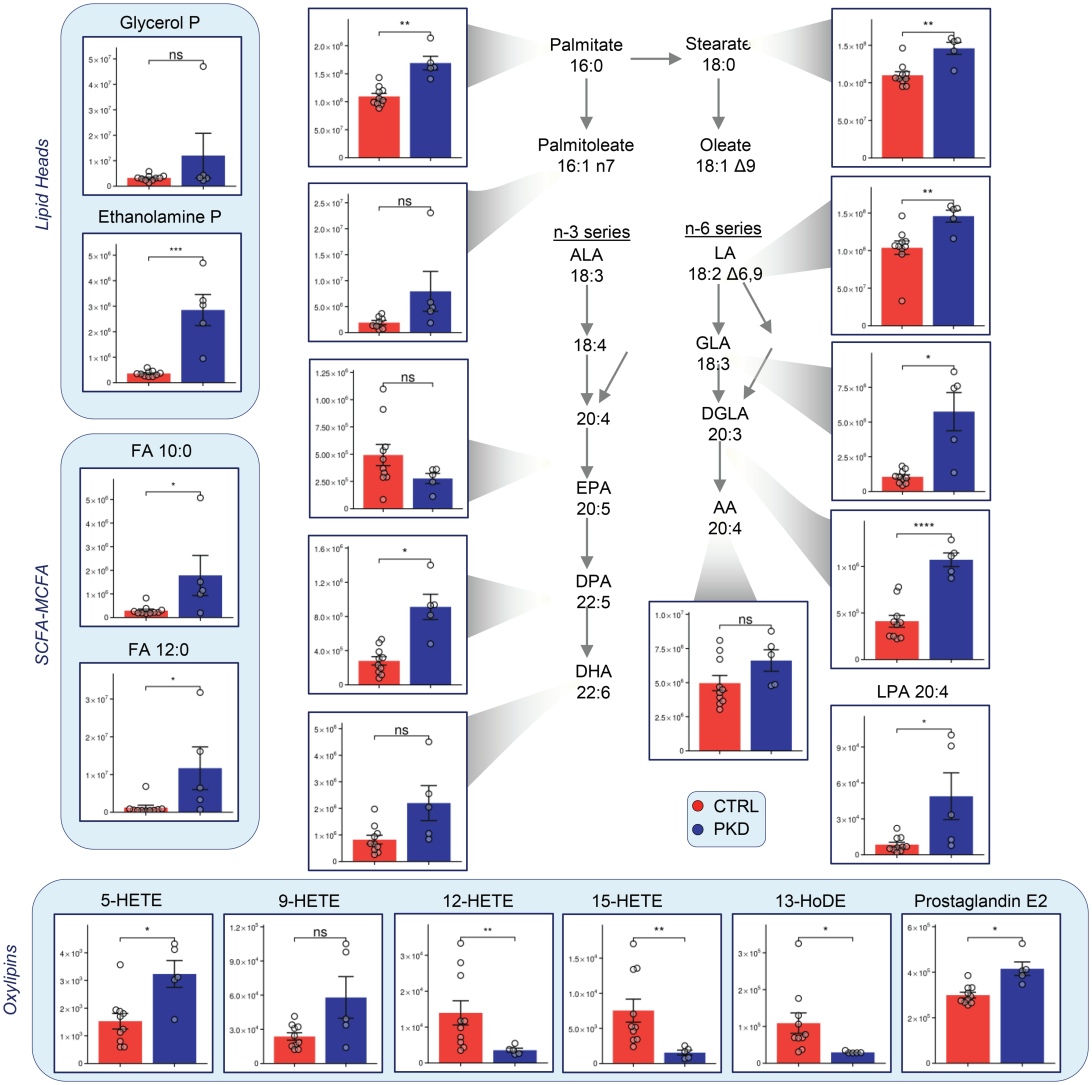
Pyruvate kinase deficiency reprograms fatty acid metabolism when compared with controls. Y-axes represent peak area (arbitrary units). Asterisks indicate significant results, error bars represent ±SEM (unpaired *t*-test, two-tailed distribution, ^*^p<0.05, ^**^p<0.01, ^***^p<0.001, and ^****^p<0.0001).

Short-chain (SCFA) medium-chain (MCFA) and long-chain fatty acids were consistently elevated in PKD samples when compared to the controls (Figure 5). Oxylipin levels varied, with 12-HETE, 15-HETE, and 13-HoDE showing lower levels in PKD patients, and 5-HETE and Prostaglandin-E2 showing higher levels.

### PKD Samples Show Metabolic Changes Characteristic of Hypoxia

Hypoxia inducible factor 1a (HIF-1a) is considered a master regulator of the cellular response to hypoxia (Iyer et al., 1998). Previous studies have shown aKG destabilizes and reduces the expression of HIF1A (Tannahill et al., 2013), and succinate (Selak et al., 2005; Majmundar et al., 2010) promotes HIF1A activation. In PKD samples, though aKG does not show significant changes, succinate is significantly elevated when compared to controls. This may suggest an increase in HIF1A activation, which has been shown to stimulate erythropoiesis to increase oxygen transport to the cells and tissues of the body (Haase, 2013).

**Figure 6:**
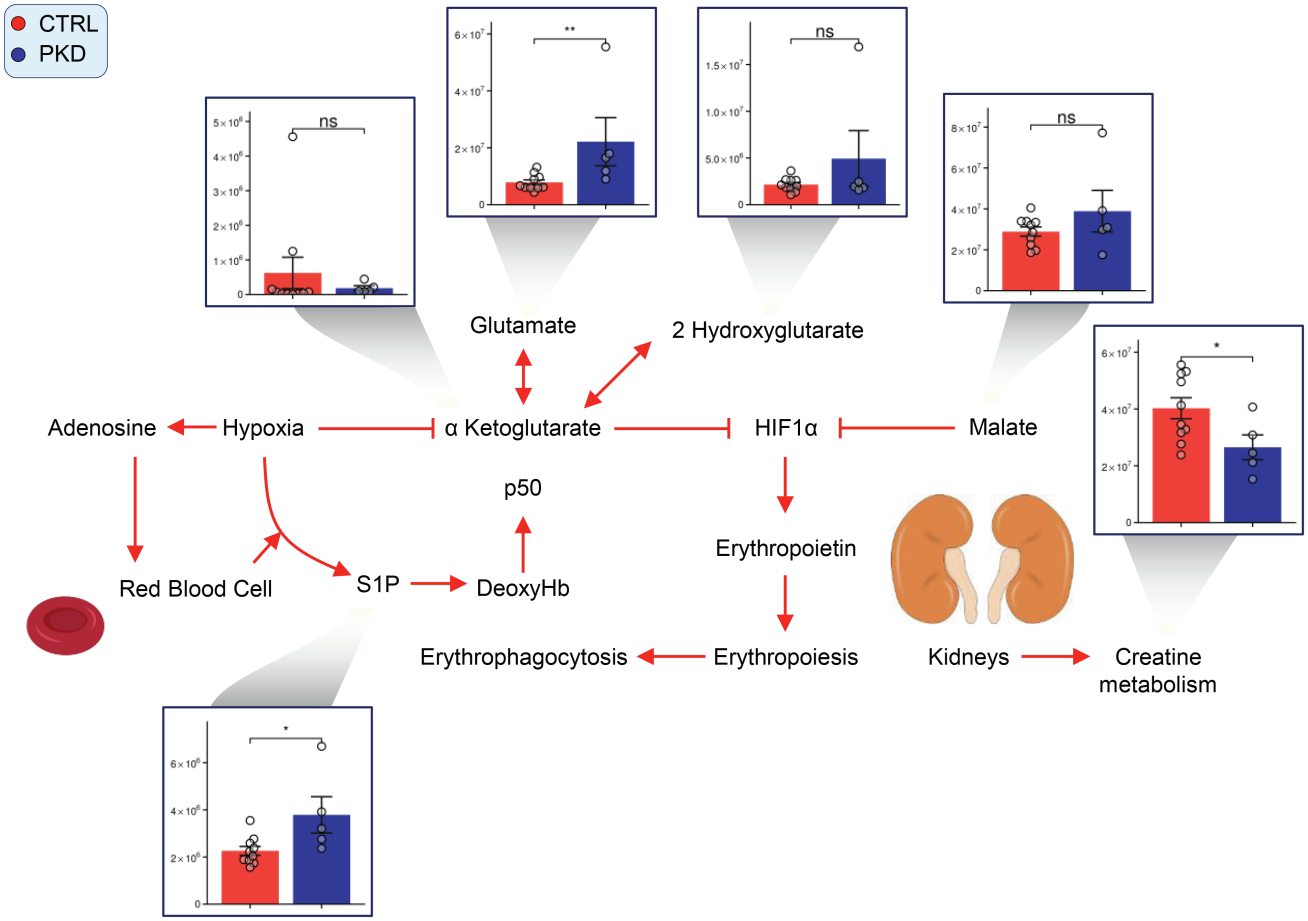
Pyruvate kinase deficiency induces a hypoxic response in red cells. Overview of hypoxia induced signaling pathways in PKD red cells. Y-axes represent peak area (arbitrary units). Asterisks indicate significant results, error bars represent ±SEM (unpaired *t*-test, two-tailed distribution, ^*^p<0.05, ^**^p<0.01, and ^***^p<0.001).

In our study, we saw elevated levels of markers of RBC response to hypoxia (Sun et al., 2016, 2017) in PKD samples, including sphingosine 1-phosphate (S1P) and 2,3-BPG (Figure 6). Interestingly, creatinine levels were lower in PKD patients. This could also reflect reduced conversion of creatine to creatinine, which is supported by the elevated creatine levels seen in PKD samples (Figure 4).

## Discussion

In the present study, we performed a pilot metabolomics analysis of RBCs from individuals with PKD. Despite the pilot nature of the study, given the limited sample size of the cohort enrolled in the test population, we care to stress the sociomedical relevance of investigation aimed at serving underrepresented minorities, such as subjects with polymorphisms of PKD genes in the Amish population (four out of five PKD subjects enrolled in this study; Rider et al., 2011). As a result, we confirm that PKD mutation R479H – the only variant investigated here – is associated with the dysregulation of late RBC glycolysis, with severe depletion of pyruvate and lactate downstream to PKD, and accumulation of metabolites upstream. Accumulation of early glycolytic intermediates and depletion of reducing equivalents may be sufficient to fuel the PPP by law of mass action, as observed for example in the context of glycolytic enzyme inhibitions by oxidant stress (e.g., oxidation of cysteine 152, 156 and histidine 179 of glyceraldehyde 3-phosphate dehydrogenase; Reisz et al., 2016) or binding to the N-terminus cytosolic domain of band 3 (Issaian et al., 2021). Indeed, steady state levels of PPP metabolites were elevated in PKD patients. This is interesting in that adaptations in RBC metabolism of PKD patients seem to be opposite to those reported in G6PD deficient subjects (Tzounakas et al., 2016; Francis et al., 2020). This observation is relevant when considering that both adaptations are deemed to have evolved to counteract the spread of malaria in regions in which this parasitic infection is endemic (Ayi et al., 2008; Mohandas and Gallagher, 2008). Though this shift can reflect increases in oxidative stress and a greater demand for GSH, increased ATP levels and comparable levels (and ratios) of GSH/GSSG in PKD samples when compared to controls suggest otherwise. Instead, the increase in the PPP may reflect a compensatory response to the decrease in late glycolytic metabolites, as the PPP produces metabolites that can reenter late glycolysis. Alternatively, this may reflect an accumulation of early glycolytic products that increase PPP activity through mass-action effects.

Despite altered glycolysis, ATP levels were surprisingly found to be higher in PKD patients in this cohort. The data is inconsistent with standard ATP measurements in the literature and previous studies on whole blood from dried blood spots (Dooijeweert et al., 2021), despite the lack of iatrogenic interventions (i.e., recent transfusion events at the time of blood draws) in any of the subjects investigated here. The high degree of reticulocytosis that accompanies of these patients may have impacted measurements of carboxylic acids (also elevated in PKD samples from earlier dried blood spot studies; Dooijeweert et al., 2021) and ATP production through residual mitochondrial activity, despite the lack of significant differences in the levels of most carboxylates between the two groups with the exception of succinate. Accumulation of succinate in PKD patients is suggestive of a potential disruption in the activity of succinate dehydrogenase (SDH) at complex II in mitochondria in other cells than RBCs, as a function of PKD-induced anemia/hypoxia, similarly to what observed in the face of pathological hemorrhagic (D’Alessandro et al., 2017) or ischemic (Chouchani et al., 2014) hypoxia in previous studies. Previous studies have hypothesized that increased glutaminolysis in PKD reticulocytes may provide an alternative form of anaplerotic carbon, compensating in the tricarboxylic acid cycle (TCA) for decreases in pyruvate from glycolysis (Van Vuren et al., 2020), and leading to reduced levels of glutamine and GSH. Though our data describes both reticulocytes and mature red cells, it revealed elevated glutamine levels and, elevated levels of its catabolites through glutaminolysis, indeed implicating an upregulation of this pathway. Levels of GSH and glutamine catabolites of other pathways were not elevated when compared to controls, further supporting this theory.

Because catabolized branched chain amino acids (BCAAs) can enter the TCA as succinyl CoA, it is not surprising that trends in BCAA levels match those of early TCA metabolites in PKD patients. C3 and C5 carnitines are considered direct products of BCAA catabolism, and C3 carnitine appeared to follow the BCAA trends (Newgard et al., 2009; Lake et al., 2015). The increase in TCA metabolites may reflect a higher proportion of reticulocytes in PKD patients, especially if some of those patients underwent splenectomy.

In the previous paragraphs, we hypothesized that increased levels of PPP metabolites in PKD patients could be at least in part explained by increases in oxidant stress in these subjects. Of note, purine oxidation products and hypoxic markers (Nemkov et al., 2018b) hypoxanthine, xanthine, and allantoate were all increased in RBCs from PKD subjects. Interestingly, previous studies on RBC storage have suggested that lower levels of these metabolites indicated higher oxidative stress (Nemkov et al., 2018b), and a compromised capacity of the transfused RBC to circulate upon transfusion (Nemkov et al., 2018b; D’Alessandro et al., 2020b).

Since purine metabolism is intertwined with methionine oxidation, purine salvage, and polyamine synthesis (Reisz et al., 2018), it is interesting to note a significant increase in several polyamines in RBCs from PKD subjects. The polyamines spermine, putrescine, and spermidine have been shown to stabilize erythrocyte membranes (Ballas et al., 1983). As such, increases in polyamines may be compensating for the decreased membrane stability characteristic of PKD associated hemolytic anemia. Polyamines also play a role in direct scavenging of reactive oxygen species, in like fashion to sulfur-containing compounds methionine (D’Alessandro et al., 2020a) and taurine (Bertolone et al., 2020), both increasing significantly in PKD subjects.

Red blood cells lack the Golgi and endoplasmic reticulum (ER) and thus cannot synthesize phospholipids *de novo* (Wu et al., 2016). Instead, they make use of the Land’s cycle to remodel oxidatively membranes, which has been shown to be upregulated under physiological (e.g., exercise stress; San-Millan et al., 2020; Nemkov et al., 2021) or pathological conditions that increase susceptibility to osmotic stress (e.g., testosterone-replacement therapy; Alexander et al., 2021; storage of RBCs from obese blood donors; Hazegh et al., 2021). Studies in RBC storage have shown the accumulation of bioactive fatty acids including HETEs, polyunsaturated fatty acids (as a function of redox-dependent fatty acid desaturase activity; Thomas et al., 2021; and oxylipins; Howie et al., 2019) – potential indicators of lipid peroxidation and increased oxidative stress (D’Alessandro et al., 2021). The accumulation of catabolites of membrane phospholipids may testify to an increased metabolism of these molecules.

Other studies have noted high levels of 2,3-BPG in PKD patients, thought to be responsible for their high tolerance of PKD associated anemia (Delivoria-Papadopoulos et al., 1969; Grace et al., 2015). In a previous study of RBCs in chronic kidney disease, S1P was demonstrated to promote 2,3-BPG production, a mechanism which appears to be active in PKD samples (Xie et al., 2020). Thus, increased levels of S1P in PKD in response to anemia increase the production of 2,3-BPG which in turn, increases oxygen offloading capabilities of RBCs. Both the potential activation of HIF1A through increased levels of succinate and the increase of S1P and subsequently 2,3-BPG levels suggest that PKD RBCs are reacting to hypoxic conditions – consistent with the observations above on increased purine breakdown and oxidation (e.g., hypoxanthine as a marker of hypoxia in cells with mitochondria).

In conclusion, in the present study, we provide the first overview of RBC metabolism in patients with PKD, expanding on previous reports on whole blood from dried blood spots (Dooijeweert et al., 2021). Though limited in scope (i.e., small cohort with a single genetic mutation R479H), the study addresses the need for basic science to investigate pathologies targeting underrepresented minorities (four out of five PKD subjects were from Amish families), with the ultimate goal to target treatments to health disparities (Hunter et al., 2021). Indeed, since these subjects suffer from anemia and therapeutic options are limited (e.g., recurring transfusions), this study could pave the way to targeted personalized investigations to drive interventions, such as supplementation of antioxidants (taurine; Bertolone et al., 2020; methionine, and choline for isoaspartyl protein damage-repair; D’Alessandro et al., 2020a); carnitine supplementation (Nikolaos et al., 2000) and dietary interventions to sustain membrane lipid remodeling in response to increased oxidant stress.

## Data Availability Statement

The original contributions presented in the study are included in the article/Supplementary Material, further inquiries can be directed to the corresponding authors.

## Ethics Statement

The studies involving human participants were reviewed and approved by IRB Pyruvate Kinase Deficiency Natural History Study Protocol No. 2014-12. The patients/participants provided their written informed consent to participate in this study.

## Author Contributions

HM and AD’A designed the study. GO and HM enrolled patients and collected the samples. MR, FG, FC, and AD’A performed metabolomics studies. AD’A prepared figures. MR and AD’A wrote the first version of the manuscript that was critically reviewed and approved by MR, FC, GO, FG, HM, and AD’A. All authors contributed to the article and approved the submitted version.

## Funding

This research was supported by funds from the National Institute of General and Medical Sciences, RM1GM131968 (AD’A), and R01HL146442 (AD’A), R01HL149714 (AD’A), R01HL148151 (AD’A), and R21HL150032 (AD’A), from the National Heart, Lung, and Blood Institute.

## Conflict of Interest

AD’A is a founder of Omix Technologies Inc. and Altis Biosciences LLC. AD’A is a consultant for Rubius Therapeutics and an advisory board member for Hemanext Inc. and FORMA Therapeutics Inc.

The remaining authors declare that the research was conducted in the absence of any commercial or financial relationships that could be construed as a potential conflict of interest.

## Publisher’s Note

All claims expressed in this article are solely those of the authors and do not necessarily represent those of their affiliated organizations, or those of the publisher, the editors and the reviewers. Any product that may be evaluated in this article, or claim that may be made by its manufacturer, is not guaranteed or endorsed by the publisher.
